# Supplementary material for: Electrically tunable layer-hybridized trions in doped WSe2 bilayers
Source: Nat Commun. 2024 Aug 7;15:6713. doi: 10.1038/s41467-024-50834-8 (PMC11306803; doi:10.1038/s41467-024-50834-8)
Supplement: Supplementary file 1 — Supplementary Information [file 41467_2024_50834_MOESM1_ESM.pdf]

Supplementary Information: Electrically tunable layer-hybridized trions in doped WSe_2 bilayers

Raul Perea-Causin,[1,2,*] Samuel Brem,[3] Fabian Buchner,[4] Yao Lu,[5] Kenji Watanabe,[6] Takashi Taniguchi,[7] John M. Lupton,[4] Kai-Qiang Lin,[4,5,†] and Ermin Malic[3,‡]

[1] *Department of Physics, Chalmers University of Technology, Gothenburg, Sweden*

[2] *Department of Physics, Stockholm University, Stockholm, Sweden*

[3] *Department of Physics, Philipps-Universität Marburg, Marburg, Germany*

[4] *Department of Physics, University of Regensburg, Regensburg, Germany*

[5] *State Key Laboratory of Physical Chemistry of Solid Surfaces, College of Chemistry and Chemical Engineering, Xiamen University, Xiamen, China*

[6] *Research Center for Electronic and Optical Materials, National Institute for Materials Science, Tsukuba, Japan*

[7] *Research Center for Materials Nanoarchitectonics, National Institute for Materials Science, Tsukuba, Japan*

[*] causin@chalmers.se

[†] kaiqiang.lin@xmu.edu.cn

[‡] ermin.malic@uni-marburg.de

CONTENTS

S1. Trion basis and Schrödinger equation 2
Onset of exciton-electron continua 5
S2. Hybrid trion photoluminescence 6
Trion-photon interaction 6
Trion-phonon interaction 6
Photoluminescence formula 7
Trion Stark effect and dipole moment 9
References 14

S1. TRION BASIS AND SCHRÖDINGER EQUATION

In the following, we sketch the derivation of the layer-hybridized trion Hamiltonian and Schrödinger equation. We start by considering the Hamiltonian of a system of interacting electrons and holes that can tunnel between two layers, $H = H_{\mathrm{eh},0} + H_{\mathrm{tun}} + H_{\mathrm{C}}$. To transform H into the trion Hamiltonian $H_{\mathrm{t},0}$ we truncate the Fock space [1] to the subspace of single trions and write the resulting three-body states in the trion basis. The terms describing free electrons and holes, $H_{\mathrm{eh},0}$, and their Coulomb interaction, H_{C}, have been discussed before in the context of monolayer trions [2], and their generalization to bilayers is straightforward. Here, we focus on the term

$$H_{\mathrm{tun}} = \sum_{l_{\mathrm{h}}\nu_{\mathrm{h}}\mathbf{k}_{\mathrm{h}}} t^{\mathrm{h}}_{\nu_{\mathrm{h}}} h^{\dagger}_{\bar{l}_{\mathrm{h}}\nu_{\mathrm{h}}\mathbf{k}_{\mathrm{h}}} h_{l_{\mathrm{h}}\nu_{\mathrm{h}}\mathbf{k}_{\mathrm{h}}} + \sum_{l_{\mathrm{e}}\nu_{\mathrm{e}}\mathbf{k}_{\mathrm{e}}} t^{\mathrm{e}}_{\nu_{\mathrm{e}}} e^{\dagger}_{\bar{l}_{\mathrm{e}}\nu_{\mathrm{e}}\mathbf{k}_{\mathrm{e}}} e_{l_{\mathrm{e}}\nu_{\mathrm{e}}\mathbf{k}_{\mathrm{e}}}, \tag{S1.1}$$

which describes the tunneling of electrons/holes from the layer $l_{\mathrm{e/h}}$ to the opposite layer, $\bar{l}_{\mathrm{e/h}}$. The tunneling strengths $t^{\mathrm{e/h}}_{\nu_{\mathrm{e/h}}}$ are assumed to be independent of the momentum $\mathbf{k}_{\mathrm{e/h}}$ within the valley $\nu_{\mathrm{e/h}}$. For naturally stacked WSe_2 bilayers, we consider only the most relevant tunneling channel, i.e. Λ-point electron tunneling with $t^{\mathrm{e}}_{\Lambda} = 0.2$ eV [3]. In the subspace of single n-type trions, the hole creation-annihilation pair $h^{\dagger}_{\bar{l}_{\mathrm{h}}\nu_{\mathrm{h}}\mathbf{k}_{\mathrm{h}}} h_{l_{\mathrm{h}}\nu_{\mathrm{h}}\mathbf{k}_{\mathrm{h}}}$ is equivalent to the projector $|h_{\bar{l}_{\mathrm{h}}\nu_{\mathrm{h}}\mathbf{k}_{\mathrm{h}}} e_{l_{\mathrm{e}1}\nu_{\mathrm{e}1}\mathbf{k}_{\mathrm{e}1}} e_{l_{\mathrm{e}2}\nu_{\mathrm{e}2}\mathbf{k}_{\mathrm{e}2}}\rangle \langle h_{l_{\mathrm{h}}\nu_{\mathrm{h}}\mathbf{k}_{\mathrm{h}}} e_{l_{\mathrm{e}1}\nu_{\mathrm{e}1}\mathbf{k}_{\mathrm{e}1}} e_{l_{\mathrm{e}2}\nu_{\mathrm{e}2}\mathbf{k}_{\mathrm{e}2}}|$, and the analogous electron operator $e^{\dagger}_{\bar{l}_{\mathrm{e}}\nu_{\mathrm{e}}\mathbf{k}_{\mathrm{e}}} e_{l_{\mathrm{e}}\nu_{\mathrm{e}}\mathbf{k}_{\mathrm{e}}}$ can be shown to correspond to two terms, $|h_{l_{\mathrm{h}}\nu_{\mathrm{h}}\mathbf{k}_{\mathrm{h}}} e_{\bar{l}_{\mathrm{e}}\nu_{\mathrm{e}}\mathbf{k}_{\mathrm{e}}} e_{l_{\mathrm{e}2}\nu_{\mathrm{e}2}\mathbf{k}_{\mathrm{e}2}}\rangle \langle h_{l_{\mathrm{h}}\nu_{\mathrm{h}}\mathbf{k}_{\mathrm{h}}} e_{l_{\mathrm{e}}\nu_{\mathrm{e}}\mathbf{k}_{\mathrm{e}}} e_{l_{\mathrm{e}2}\nu_{\mathrm{e}2}\mathbf{k}_{\mathrm{e}2}}|$ and $|h_{l_{\mathrm{h}}\nu_{\mathrm{h}}\mathbf{k}_{\mathrm{h}}} e_{l_{\mathrm{e}1}\nu_{\mathrm{e}1}\mathbf{k}_{\mathrm{e}1}} e_{\bar{l}_{\mathrm{e}}\nu_{\mathrm{e}}\mathbf{k}_{\mathrm{e}}}\rangle \langle h_{l_{\mathrm{h}}\nu_{\mathrm{h}}\mathbf{k}_{\mathrm{h}}} e_{l_{\mathrm{e}1}\nu_{\mathrm{e}1}\mathbf{k}_{\mathrm{e}1}} e_{l_{\mathrm{e}}\nu_{\mathrm{e}}\mathbf{k}_{\mathrm{e}}}|$, provided that double counting is avoided.

Now we expand the three-particle states in trion basis, $|h_{l_\mathrm{h}\nu_\mathrm{h}\mathbf{k}_\mathrm{h}} e_{l_{\mathrm{e}1}\nu_{\mathrm{e}1}\mathbf{k}_{\mathrm{e}1}} e_{l_{\mathrm{e}2}\nu_{\mathrm{e}2}\mathbf{k}_{\mathrm{e}2}}\rangle = \sum_\lambda \psi^\lambda_{\nu l,\mathbf{k}_1\mathbf{k}_2} |T^\lambda_{\nu,\mathbf{Q}}\rangle$, where ν and l are the trion compound valley and layer indices, λ is the trion state index, $\mathbf{k}_1$ and $\mathbf{k}_2$ are the relative electron-hole momenta, and $\mathbf{Q}$ is the center-of-mass trion momentum. Then we obtain $H_\mathrm{tun} = \sum_n \psi^\lambda_{\nu l,\mathbf{k}_1\mathbf{k}_2} \mathcal{H}^{(\mathrm{tun})}_{\nu ll'} (\psi^{\lambda'}_{\nu l',\mathbf{k}_1\mathbf{k}_2})^* |T^\lambda_{\nu,\mathbf{Q}}\rangle \langle T^{\lambda'}_{\nu,\mathbf{Q}}|$, where the sum is taken over $n = \{\lambda, \lambda', l, l', \nu, \mathbf{Q}, \mathbf{k}_1, \mathbf{k}_2\}$ and we have introduced

$$\mathcal{H}^{(\mathrm{tun})}_{ll'} = t^\mathrm{e} \delta_{\bar{l}_{\mathrm{e}1},l'_{\mathrm{e}1}} \delta_{l_{\mathrm{e}2},l'_{\mathrm{e}2}} \delta_{l_\mathrm{h},l'_\mathrm{h}} + t^\mathrm{e} \delta_{l_{\mathrm{e}1},l'_{\mathrm{e}1}} \delta_{\bar{l}_{\mathrm{e}2},l_{\mathrm{e}2}} \delta_{l_\mathrm{h},l'_\mathrm{h}} + t^\mathrm{h} \delta_{l_{\mathrm{e}1},l'_{\mathrm{e}1}} \delta_{l_{\mathrm{e}2},l'_{\mathrm{e}2}} \delta_{\bar{l}_\mathrm{h},l'_\mathrm{h}} \tag{S1.2}$$

which depends implicitly on the valley configuration ν. Furthermore, we exploit the equivalence between the projector $|T\rangle\langle T|$ and the operator pair $T^\dagger T$ at low trion densities. After applying the same procedure to the free and interaction terms, $H_{\mathrm{eh},0}$ and H_C, the trion Schrödinger equation in momentum space emerges naturally in H and the trion Hamiltonian $H_{\mathrm{t},0} = \sum_{\lambda\nu\mathbf{Q}} E^\mathrm{t}_{\lambda\nu\mathbf{Q}} T^\dagger_{\lambda\nu\mathbf{Q}} T_{\lambda\nu\mathbf{Q}}$ is obtained. The minimum of the hybrid trion dispersion—which is energetically distant from the avoided crossing between hybrid bands—retains its parabolic form, $E^\mathrm{t}_{\lambda\nu\mathbf{Q}} = E^\mathrm{t}_{\lambda\nu 0} + \hbar^2\mathbf{Q}^2/2M_{\lambda\nu}$, with the effective trion mass given by $M^{-1}_{\lambda\nu} = \sum_l |w^\lambda_{\nu l}|^2 \tilde{M}^{-1}_{\nu l}$. Here, $w^\lambda_{\nu l}$ is the trion layer mixing coefficient and $\tilde{M}_{\nu l}$ is the effective mass of the unperturbed trion with the compound layer index $l = \{l_\mathrm{h}, l_{\mathrm{e}1}, l_{\mathrm{e}2}\}$. The real-space trion wave function $\Psi^\lambda_{\nu l}(\mathbf{r}_1,\mathbf{r}_2) = \sum_{\mathbf{k}_1\mathbf{k}_2} \mathrm{e}^{i\mathbf{k}_1\cdot\mathbf{r}_1} \mathrm{e}^{i\mathbf{k}_2\cdot\mathbf{r}_2} \psi^\lambda_{\nu l,\mathbf{k}_1\mathbf{k}_2}$ satisfies the Schrödinger equation,

$$\sum_{l'} \mathcal{H}_{\nu ll'}(\mathbf{r}_1,\mathbf{r}_2) \Psi^\lambda_{\nu l'}(\mathbf{r}_1,\mathbf{r}_2) = E^\mathrm{t}_{\lambda\nu 0} \Psi^\lambda_{\nu l}(\mathbf{r}_1,\mathbf{r}_2), \tag{S1.3}$$

We now drop the valley index ν for simplicity. The electron-electron-hole Hamiltonian reads $\mathcal{H}_{ll'}(\mathbf{r}_1,\mathbf{r}_2) = \left[\mathcal{H}^{(0)}_l(\mathbf{r}_1,\mathbf{r}_2) + \mathcal{H}^{(\mathrm{C})}_l(\mathbf{r}_1,\mathbf{r}_2)\right] \delta_{ll'} + \mathcal{H}^{(\mathrm{tun})}_{ll'}$. Here, the free term

$$\mathcal{H}^{(0)}_l(\mathbf{r}_1,\mathbf{r}_2) = \tilde{E}^\mathrm{t}_l - \frac{\hbar^2\nabla^2_{\mathbf{r}_1}}{2\mu_{l_\mathrm{h}l_{\mathrm{e}1}}} - \frac{\hbar^2\nabla^2_{\mathbf{r}_2}}{2\mu_{l_\mathrm{h}l_{\mathrm{e}2}}} - \frac{\hbar^2\nabla_{\mathbf{r}_1}\cdot\nabla_{\mathbf{r}_2}}{\tilde{m}_{l_\mathrm{h}}} \tag{S1.4}$$

is given by $\tilde{E}^\mathrm{t}_l = \tilde{E}^\mathrm{h}_{l_\mathrm{h}} + \tilde{E}^\mathrm{e}_{l_{\mathrm{e}1}} + \tilde{E}^\mathrm{e}_{l_{\mathrm{e}2}}$, i.e. the sum of the unperturbed single-particle band-edge energies, and by the kinetic energy of the relative electron-hole motion. The latter is determined by the effective hole mass $\tilde{m}_{l_\mathrm{h}}$ and the reduced electron-hole masses given by $\mu^{-1}_{l_\mathrm{h}l_n} = \tilde{m}^{-1}_{l_\mathrm{h}} + \tilde{m}^{-1}_{l_n}$. The external out-of-plane electric field ε is incorporated into the model via the shift of the single-particle energies, $\tilde{E}^{\mathrm{e/h}}_{l_n} \to \tilde{E}^{\mathrm{e/h}}_{l_n} \pm \sigma_{l_n} e_0 d\varepsilon/2$, with $+$ $(-)$ for electrons (holes), $\sigma_\mathrm{top} = +1$, $\sigma_\mathrm{bottom} = -1$, the elementary charge e_0, and the layer separation $d = 0.65$ nm [4].

The Coulomb interaction between the trion's constituent particles is described by

$$\mathcal{H}^{(\mathrm{C})}_l(\mathbf{r}_1,\mathbf{r}_2) = V_{l_{\mathrm{e}1}l_{\mathrm{e}2}}(\mathbf{r}_2 - \mathbf{r}_1) - V_{l_\mathrm{h}l_{\mathrm{e}1}}(\mathbf{r}_1) - V_{l_\mathrm{h}l_{\mathrm{e}2}}(\mathbf{r}_2), \tag{S1.5}$$

where the potential for intra ($V_{l=l'}$) and interlayer ($V_{l\neq l'}$) interactions is modeled following the generalization of the Rytova-Keldysh potential [5, 6] to bilayer systems [7] with dielectric constants from Refs. [4, 8].

For each possible valley configuration, the energetically lowest trion state is obtained by minimizing the trion energy in Eq. (S1.3) with the wave function ansatz [2]

$$\Psi_l(\mathbf{r}_1, \mathbf{r}_2) = \frac{w_l}{\mathcal{N}_l}\left(\mathrm{e}^{-\frac{|\mathbf{r}_1|}{a_{1,l}}-\frac{|\mathbf{r}_2|}{a_{2,l}}} + C_l\mathrm{e}^{-\frac{|\mathbf{r}_1|}{b_{1,l}}-\frac{|\mathbf{r}_2|}{b_{2,l}}}\right), \tag{S1.6}$$

or simply $\Psi_l(\mathbf{r}_1, \mathbf{r}_2) = w_l\psi_l(\mathbf{r}_1, \mathbf{r}_2)$, with the variational parameters $w_l, a_{1,l}, a_{2,l}, b_{1,l}, b_{2,l}, C_l$ depending implicitly on the spin-valley index ν and with the normalization factor $\mathcal{N}_l$ ensuring $\iint d^2\mathbf{r}_1 d^2\mathbf{r}_2 |\psi_l(\mathbf{r}_1, \mathbf{r}_2)|^2 = 1$. We have dropped λ from the notation as this ansatz only yields the energetically lowest state. Equation (S1.6) describes the trion as a hybrid of states with a different layer configuration l. The contribution from each layer configuration is weighted by the coefficient w_l and described by the function ψ_l, which considers the superposition between two states with different electron-hole radii $a_{1/2,l}$ and $b_{1/2,l}$ weighted by C_l. This wave function thus allows to consider an imbalance in the effective mass of the two electrons, i.e. $m_{l_{\mathrm{e1}}} \neq m_{l_{\mathrm{e2}}}$, or in the electron-hole interaction, i.e. $V_{l_\mathrm{h},l_{\mathrm{e1}}} \neq V_{l_\mathrm{h},l_{\mathrm{e2}}}$, resulting in the reduction of the trion binding energy [2]. Note that $\Psi_l(\mathbf{r}_1, \mathbf{r}_2)$ is formally an envelope function that is generally asymmetric with the permutation $\mathbf{r}_1 \leftrightarrow \mathbf{r}_2$ [9], whereas the fermionic antisymmetry is contained in the trion creation/annihilation operators [2].

We first obtain the lowest unbound exciton-electron state by global optimization (differential evolution) followed by local optimization (Nelder-Mead with relative tolerance 10^{-7}). The obtained layer mixing coefficients are then taken as initial parameters to minimize the trion energy with the same local optimization algorithm. Furthermore, we have confirmed the global convergence of the trion energies for the relevant states discussed in the manuscript. In addition, we have checked that the disregarded hole tunneling would only result in a 2% mixing with the additional layer. The calculated trion energies, trion binding energies, and electron layer probabilities are summarized in Table S1 for the relevant states discussed throughout the manuscript. The electron's probability to be in the top layer is computed as $p_\mathrm{t}^\mathrm{e} = \sum_l |w_l|^2$, where the sum is restricted to $l = \{\mathrm{t}, l_{\mathrm{h1}}, l_{\mathrm{h2}}\}$ for p-type trions and $l = \{l_\mathrm{h}, \mathrm{t}, l_{\mathrm{e2}}\}$ or $l = \{l_\mathrm{h}, l_{\mathrm{e1}}, \mathrm{t}\}$ for n-type.

State	$E^{\rm t}$ (meV)	$E^{\text{x-e}}$-$E^{\rm t}$ (meV)	$p^{\rm e}_{\rm t}$
$X^+_{\Lambda tt}$	0	7 (7)	0.82
$X^+_{\Lambda tb}$	5	2 (45)	0.80
$X^+_{\Lambda' tt}$	48	1 (1)	0.35
X^+_{Ktt}	135	9 (9)	1.00
$X^-_{t\Lambda\Lambda}$	0	12 (12)	0.77 (0.77)
$X^-_{t\Lambda\Lambda'}$	12	0 (42)	0.82 (0.26)
$X^-_{t\Lambda'\Lambda'}$	50	4 (4)	0.31 (0.31)
$X^-_{tK\Lambda}$	130	20 (5)	1.00 (0.79)
$X^-_{tK\Lambda'}$	146	2 (31)	1.00 (0.26)

TABLE S1. Trion energies relative to the ground state ($E^{\rm t}$), trion binding energies ($E^{\text{x-e}}$-$E^{\rm t}$), and probability that the electron is in the top layer ($p^{\rm e}_{\rm t}$). The trion binding energy with respect to the exciton formed by the second equal charge is denoted in parenthesis. For n-type trions, the top-layer probability for the second electron is also denoted in parenthesis. All the numbers reported are in the absence of an electric field.

Onset of exciton-electron continua

In order to consistently identify the binding energies of different trion states, we establish the energetic offset of trion continua by solving the eigenvalue problem of a non-interacting layer-hybridized exciton-electron compound,

$$\mathcal{H}_l^{(\text{x-e},0)}(\mathbf{r})\chi_l(\mathbf{r}) + \sum_{l'} \mathcal{H}_{ll'}^{(\text{tun})}\chi_{l'}(\mathbf{r}) = E^{\text{x-e}}\chi_l(\mathbf{r}), \tag{S1.7}$$

with $\mathcal{H}_l^{(\text{x-e},0)}(\mathbf{r}) = \tilde{E}^{\rm t}_l - \hbar^2\nabla^2_{\mathbf{r}}/2\mu_{l_{\rm h}l_{\rm e1}} - V_{l_{\rm h}l_{\rm e1}}(\mathbf{r})$. Here we have focused on the onset of the trion continuum, i.e. the energetically lowest state where the free electron has zero kinetic energy. We minimize the energy $E^{\text{x-e}}$ with the variational hydrogenic wavefunction $\chi_l(\mathbf{r}) = \tilde{w}_l\sqrt{2/\pi a_l^2}\exp(-|\mathbf{r}|/a_l)$, where a_l and $\tilde{w}_l$ are the variational parameters describing the exciton Bohr radius and layer mixing, respectively. We perform this procedure considering the case where the electron with layer index $l_{\rm e1}$ is bound to the hole, and then considering the opposite case simply by exchanging the indices $l_{\rm e1} \leftrightarrow l_{\rm e2}$ in Eq. (S1.7)

S2. HYBRID TRION PHOTOLUMINESCENCE

Trion-photon interaction

By exploiting the approach outlined in Section S1, one can derive the Hamiltonian describing the trion-photon interaction,

$$H_{\text{t-phot}} = \sum_{\lambda\nu\eta\nu_{\mathrm{e}}\mathbf{Q}\boldsymbol{\kappa}} \mathcal{M}^{\lambda\eta,\nu\nu_{\mathrm{e}}}_{\mathbf{Q}\boldsymbol{\kappa}} c^\dagger_{\boldsymbol{\kappa}} e^\dagger_{\eta\nu_{\mathrm{e}}\mathbf{Q}-\boldsymbol{\kappa}_\parallel} T_{\lambda\nu\mathbf{Q}} + \text{H.c.}, \tag{S2.1}$$

where the first term describes processes where a trion in state λ with spin-valley index ν and momentum $\mathbf{Q}$ is annihilated ($T_{\lambda\nu\mathbf{Q}}$), while a photon with momentum $\boldsymbol{\kappa}$ and an electron in the layer-hybridized state η, at valley ν_{e}, and with momentum $\mathbf{Q}-\boldsymbol{\kappa}_\parallel$ are created ($c^\dagger_{\boldsymbol{\kappa}} e^\dagger_{\eta\nu_{\mathrm{e}}\mathbf{Q}-\boldsymbol{\kappa}_\parallel}$). The opposite process is described by the Hermitian conjugate. The trion-photon matrix element reads $\mathcal{M}^{\lambda\eta,\nu\nu_{\mathrm{e}}}_{\mathbf{Q}\boldsymbol{\kappa}} = \sum_{ll_{\mathrm{e}}} \tilde{\mathcal{M}}^{ll_{\mathrm{e}},\nu\nu_{\mathrm{e}}}_{\mathbf{Q}\boldsymbol{\kappa}} (w^\lambda_{\nu l})^* B^\eta_{\nu_{\mathrm{e}} l_{\mathrm{e}}}$, with the coupling strength in the layer basis [2],

$$\begin{aligned}\tilde{\mathcal{M}}^{ll_{\mathrm{e}},\nu\nu_{\mathrm{e}}}_{\mathbf{Q}\boldsymbol{\kappa}} =& \mathcal{M}^{l_{\mathrm{h}},\nu_{\mathrm{h}}}_{\boldsymbol{\kappa}} \Bigg[\mathcal{D}_{ll_{\mathrm{e}},\nu\nu_{\mathrm{e}}} \int d^2\mathbf{r}\ \mathrm{e}^{i\alpha_{\nu l}\mathbf{Q}\cdot\mathbf{r}} \psi_{\nu l}(0,\mathbf{r}) \\ & -\mathcal{D}'_{ll_{\mathrm{e}},\nu\nu_{\mathrm{e}}} \int d^2\mathbf{r}\ \mathrm{e}^{i\alpha'_{\nu l}\mathbf{Q}\cdot\mathbf{r}} \psi_{\nu l}(\mathbf{r},0)\Bigg],\end{aligned} \tag{S2.2}$$

which includes the intralayer electron-photon matrix element $\mathcal{M}^{l_{\mathrm{h}},\nu_{\mathrm{h}}}_{\boldsymbol{\kappa}}$ [10, 11]. Here, we have considered only intralayer recombination, as it is orders of magnitude more efficient than interlayer processes [12, 13]. The selection rules thus read $\mathcal{D}^{(\prime)}_{ll_{\mathrm{e}},\nu\nu_{\mathrm{e}}} = \delta_{l_{\mathrm{h}},l_{\mathrm{e}1(2)}} \delta_{\nu_{\mathrm{h}},\nu_{\mathrm{e}1(2)}} \delta_{l_{\mathrm{e}},l_{\mathrm{e}2(1)}} \delta_{\nu_{\mathrm{e}},\nu_{\mathrm{e}2(1)}}$, i.e. the same valley and layer of the recombining electron and hole is enforced, and the remaining electron remains in the same valley and layer. We have further introduced the mass ratio $\alpha^{(\prime)}_{\nu l} = (\tilde{m}_{\nu_{\mathrm{h}} l_{\mathrm{h}}} + \tilde{m}_{\nu_{\mathrm{e}1(2)} l_{\mathrm{e}1(2)}})/\tilde{M}_\nu$, with the total trion mass $M_{\nu l} = \tilde{m}_{\nu_{\mathrm{h}} l_{\mathrm{h}}} + \tilde{m}_{\nu_{\mathrm{e}1} l_{\mathrm{e}1}} + \tilde{m}_{\nu_{\mathrm{e}2} l_{\mathrm{e}2}}$. The electron layer-mixing coefficient $B^\eta_{\nu_{\mathrm{e}} l_{\mathrm{e}}}$ and energy $E^{\mathrm{e}}_{\eta\nu_{\mathrm{e}}\mathbf{k}} = E^{\mathrm{e}}_{\eta\nu_{\mathrm{e}}0} + \hbar^2\mathbf{k}^2/2m^{\mathrm{e}}_{\eta\nu_{\mathrm{e}}}$ are determined by the tunneling eigenvalue problem $\tilde{E}^{\mathrm{e}}_{\nu_{\mathrm{e}} l_{\mathrm{e}}} B^\eta_{\nu_{\mathrm{e}} l_{\mathrm{e}}} + t^{\mathrm{e}}_\nu B^\eta_{\nu_{\mathrm{e}} \bar{l}_{\mathrm{e}}} = E^{\mathrm{e}}_{\eta\nu_{\mathrm{e}}0} B^\eta_{\nu_{\mathrm{e}} l_{\mathrm{e}}}$ and the effective mass $(m^{\mathrm{e}}_{\eta\nu_{\mathrm{e}}})^{-1} = \sum_{l_{\mathrm{e}}} |B^\eta_{\nu_{\mathrm{e}} l_{\mathrm{e}}}|^2 (\tilde{m}^{\mathrm{e}}_{\nu_{\mathrm{e}} l_{\mathrm{e}}})^{-1}$.

Trion-phonon interaction

The trion-phonon interaction Hamiltonian, which is also derived with the approach outlined in Section S1, reads

$$H_{\text{t-phon}} = \sum_{\lambda\lambda'\nu\nu'\mathbf{Q}\mathbf{q}} G^{\lambda\lambda',\nu\nu'}_{\mathbf{q}} T^\dagger_{\lambda'\nu'\mathbf{Q}+\mathbf{q}} T_{\lambda\nu\mathbf{Q}} \left(b_{\mathbf{q}} + b^\dagger_{-\mathbf{q}}\right), \tag{S2.3}$$

which is determined by the trion-phonon matrix element $G_{\mathbf{q}}^{\lambda\lambda',\nu\nu'} = \sum_l \tilde{G}_{\mathbf{q}}^{ll,\nu\nu'} (w_{\nu l}^{\lambda})^* w_{\nu' l}^{\lambda'}$. The matrix element in layer basis reads [2]

$$\begin{aligned}\tilde{G}_{\mathbf{q}}^{ll,\nu\nu'} &= \delta_{\nu_{e1},\nu'_{e1}} \delta_{\nu_{e2},\nu'_{e2}} g^{\mathrm{h}}_{l_{\mathrm{h}},\nu_{\mathrm{h}}\nu'_{\mathrm{h}},\mathbf{q}} \mathcal{F}^{ll,\nu\nu'}_{-\beta_{\nu l}\mathbf{q},-\beta'_{\nu l}\mathbf{q}} \\ &+ \delta_{\nu_{\mathrm{h}},\nu'_{\mathrm{h}}} \delta_{\nu_{e2},\nu'_{e2}} g^{\mathrm{e}}_{l_{e1},\nu_{e1}\nu'_{e1},\mathbf{q}} \mathcal{F}^{ll,\nu\nu'}_{\alpha'_{\nu l}\mathbf{q},-\beta'_{\nu l}\mathbf{q}} \\ &+ \delta_{\nu_{\mathrm{h}},\nu'_{\mathrm{h}}} \delta_{\nu_{e1},\nu'_{e1}} g^{\mathrm{e}}_{l_{e2},\nu_{e2}\nu'_{e2},\mathbf{q}} \mathcal{F}^{ll,\nu\nu'}_{-\beta_{\nu l}\mathbf{q},\alpha_{\nu l}\mathbf{q}} \end{aligned} \quad \text{(S2.4)}$$

with $\beta_{\nu l}^{(\prime)} = \tilde{m}_{\nu_{e1(2)} l_{e1(2)}} / \tilde{M}_\nu$ and the form factor $\mathcal{F}_{\mathbf{q},\mathbf{q}'}^{ll,\nu\nu'} = \iint d^2\mathbf{r}_1 d^2\mathbf{r}_2 \, \mathrm{e}^{i\mathbf{q}\cdot\mathbf{r}_1} \mathrm{e}^{i\mathbf{q}'\cdot\mathbf{r}_2} \psi_{\nu l}^*(\mathbf{r}_1,\mathbf{r}_2) \psi_{\nu' l}(\mathbf{r}_1,\mathbf{r}_2)$. Here, we have assumed that the effective masses in the initial and final states are similar and thus neglected additional momentum transfers due to unequal masses. In the case where the effective masses are different, we take the average of $|\tilde{G}_{\mathbf{q}}^{ll,\nu\nu'}|^2$ and $|\tilde{G}_{\mathbf{q}}^{ll,\nu'\nu}|^2$ to enforce the symmetry of the matrix element. Moreover, spin-flip transitions are disregarded as they are generally weaker than spin-conserving ones [14, 15]. We have introduced in Eq. (S2.3) the annihilation (creation) operator $b_{\mathbf{q}}^{(\dagger)}$ acting on a phonon with momentum $\mathbf{q}$, and with the mode and valley being implicitly contained in the same index. The phonon energies and deformation potentials for longitudinal/transversal acoustic/optical (LA, TA, LO, TO) and the out-of-plane A_1 mode around the high symmetry points of the Brillouin zone are described with ab initio input parameters from Ref. [16].

Photoluminescence formula

After having introduced the trion-photon and trion-phonon interactions as well as the electron tunneling eigenvalue problem, we briefly describe the derivation of the PL formula and provide additional numerical details.

The full Hamiltonian of the system reads $H = H_0 + H_{\text{t-phot}} + H_{\text{t-phon}}$, with $H_0 = H_{\mathrm{t},0} + H_{\mathrm{e},0} + H_{\mathrm{phot},0} + H_{\mathrm{phon},0}$, where we have now introduced $H_{\mathrm{e},0} = \sum_{\eta\nu_e\mathbf{k}} E^{\mathrm{e}}_{\eta\nu_e\mathbf{k}} e^\dagger_{\eta\nu_e\mathbf{k}} e_{\eta\nu_e\mathbf{k}}$, $H_{\mathrm{phot},0} = \sum_{\boldsymbol{\kappa}} \hbar\omega_{\boldsymbol{\kappa}} c^\dagger_{\boldsymbol{\kappa}} c_{\boldsymbol{\kappa}}$, and $H_{\mathrm{phon},0} = \sum_{\mathbf{q}} \hbar\Omega_{\mathbf{q}} b^\dagger_{\mathbf{q}} b_{\mathbf{q}}$ accounting for layer-hybridized electrons as well as phonons and photons with energies $E^{\mathrm{e}}_{\eta\nu_e\mathbf{k}}$, $\hbar\omega_{\boldsymbol{\kappa}}$, and $\hbar\Omega_{\mathbf{q}}$, respectively. The PL intensity is given by the photon emission rate, i.e. $I_{\mathrm{PL}}(\omega) = \sum_{\boldsymbol{\kappa}} \delta(\omega - \omega_{\boldsymbol{\kappa}}) \dot{n}_{\boldsymbol{\kappa}}$, where $n_{\boldsymbol{\kappa}} = \langle c^\dagger_{\boldsymbol{\kappa}} c_{\boldsymbol{\kappa}} \rangle$ is the photon number. Exploiting the Heisenberg equation within the cluster expansion and truncation scheme [2, 17, 18] yields a closed set of equations coupling $n_{\boldsymbol{\kappa}}$ to the correlations $\langle c^\dagger_{\boldsymbol{\kappa}} e^\dagger_{\eta\nu_e\mathbf{Q}} T_{\lambda\nu\mathbf{Q}} \rangle^{\mathrm{c}}$, $\langle c^\dagger_{\boldsymbol{\kappa}} e^\dagger_{\eta\nu_e\mathbf{Q}} T_{\lambda\nu\mathbf{Q}-\mathbf{q}} b^{(\dagger)}_{\mp\mathbf{q}} \rangle^{\mathrm{c}}$ and the trion occupation $N_{\lambda\nu\mathbf{Q}} = \langle T^\dagger_{\lambda\nu\mathbf{Q}} T_{\lambda\nu\mathbf{Q}} \rangle$, which is well described by a Boltzmann distribution at sufficiently low densities. Solving this set

of equations in the steady state yields the PL formula for dark and bright layer-hybridized trions,

$$I_{\mathrm{PL}}(\omega) = \frac{2}{\hbar} \sum_{\lambda\eta\nu\nu_{\mathrm{e}}\mathbf{Q}} \frac{\left|\mathcal{M}_{\mathbf{Q}}^{\lambda\eta,\nu\nu_{\mathrm{e}}}\right|^2}{(E^{\mathrm{t}}_{\lambda\nu\mathbf{Q}} - E^{\mathrm{e}}_{\eta\nu_{\mathrm{e}}\mathbf{Q}} - \hbar\omega)^2 + (\gamma^{\mathrm{t}}_{\lambda\nu\mathbf{Q}})^2} \times \left[N_{\lambda\nu\mathbf{Q}}\gamma^{\text{t-phot}}_{\lambda\nu\mathbf{Q}} + \sum_{\lambda'\nu'\mathbf{q}\pm} \frac{N_{\lambda'\nu'\mathbf{Q}+\mathbf{q}} \left|G_{\mathbf{q}}^{\lambda\lambda',\nu\nu'}\right|^2 \rho^{\pm}_{\mathbf{q}} \gamma^{\mathrm{t}}_{\lambda'\nu'\mathbf{Q}+\mathbf{q}}}{\left(\Delta_{\mathbf{Q}\mathbf{q}}^{\lambda'\eta\nu'\nu_{\mathrm{e}}}\right)^2 + \left(\gamma^{\mathrm{t}}_{\lambda'\nu'\mathbf{Q}+\mathbf{q}}\right)^2} \right]. \quad \text{(S2.5)}$$

The first term describes the direct recombination of bright trions via the electron recoil effect, where the electron-hole pair in the trion recombines by emitting a photon and the trion momentum $\mathbf{Q}$ is transferred to the remaining electron [19, 20]. Here, $E^{\mathrm{e}}_{\eta\nu_{\mathrm{e}}\mathbf{Q}}$ is the energy of an electron in the layer-hybridized single-particle state η. Similar to the ARPES signatures of hybrid excitons [21], the PL arising from a particular trion state should give rise to two PL peaks separated by the energy difference between the two hybrid electron bands η acting as the possible final states. In the considered system, however, the relative intensity between the two peaks vanishes due to the marginal Coulomb-induced mixing between the hybrid electron bands forming the trion—a consequence of the trion binding energy being small compared to the large energetic offset between the hybrid electron bands.

The second term in Eq. (S2.5) accounts for phonon-assisted recombination, where a dark trion scatters with a phonon into a virtual bright state and then recombines, leaving behind the additional electron. Here, we have introduced the energy offset $\Delta_{\mathbf{Q}\mathbf{q}}^{\lambda'\eta\nu'\nu_{\mathrm{e}}} = E^{\mathrm{t}}_{\lambda'\nu'\mathbf{Q}+\mathbf{q}} - E^{\mathrm{e}}_{\eta\nu_{\mathrm{e}}\mathbf{Q}} - \hbar\omega \mp \hbar\Omega_{\mathbf{q}}$, and $\rho^{\pm}_{\mathbf{q}} = n_{\mathbf{q}} + (1 \pm 1)/2$ with the phonon number $n_{\mathbf{q}}$.

The dephasing $\gamma^{\mathrm{t}} = \gamma^{\text{t-phot}} + \gamma^{\text{t-phon}}$ is described on a phenomenological level with the experimentally realistic parameters [22] $\gamma^{\text{t-phot}} = 1$ meV and $\gamma^{\text{t-phon}} = 1\ \text{meV} + 3 \cdot (2.5\ \mu\text{eV/K})\ T$, where T is the lattice temperature and the factor 3 accounts for the microscopically-determined stronger trion-phonon scattering compared to exciton-phonon scattering [23]. Finally, to stay consistent with the long-wavelength approximation considered around each symmetry point for the phonon dispersion and deformation potentials, we introduce a cutoff in the phonon momentum, $|\mathbf{q}| < 0.1\ \text{nm}^{-1}$, chosen to match the asymmetric broadening of experimentally measured phonon sidebands of dark trions [24]. The inclusion of this cutoff does not qualitatively alter the results nor the main message of the work, i.e. the dominant role of layer-hybridized dark trions and the electrical tunability of their PL signatures. Omitting the momentum cutoff would only result in broader low-energy tails of the phonon

sidebands and a slightly altered bright/dark PL intensity ratio.

To reduce the numerical cost in the electrical tunability study, we calculate the trion energies and variational parameters at electric fields $\varepsilon = -0.2, -0.1, 0.0, 0.1, 0.2$ V/nm and interpolate these quantities at intermediate values with a fourth-order polynomial. Moreover, we run the calculations for trion states with one hole in the top layer and extract the states with opposite configuration by exploiting the symmetry of the system with respect to the electric field, e.g. the wave function and energy of $\mathrm{X}^-_{\mathrm{b}\Lambda'\Lambda'}$ for $\varepsilon = 0.2$ V/nm are equal to those of $\mathrm{X}^-_{\mathrm{t}\Lambda\Lambda}$ for $\varepsilon = -0.2$ V/nm. Furthermore, to consistently predict changes in the PL peak intensity we consider a constant trion density of $10^{11}\mathrm{cm}^{-2}$ and take into account the occupation of excited states including the trion continuum assuming a Saha-like thermodynamic equilibrium [25, 26].

Trion Stark effect and dipole moment

The trion PL resonances are given by $\hbar\omega = E^{\mathrm{t}} - E^{\mathrm{e}}$ (with an additional $-\hbar\Omega$ for phonon sidebands), where we have omitted valley and momentum indices for simplicity and assumed that the free electron is in the lowest layer-hybridized state. It can be seen from the trion and electron eigenvalue problems introduced above that, when an electric field ε is applied, the trion and electron energies are shifted by $E^{\mathrm{t}} \to E^{\mathrm{t}} + \sum_n (\pm 1) e_0 d^{(n)}_{\mathrm{avg,t}}\varepsilon$ and $E^{\mathrm{e}} \to E^{\mathrm{e}} + e_0 d_{\mathrm{avg,e}}\varepsilon$, with $+1$ for electrons and -1 for holes. We have introduced here the average positions for the trion's constituent particles ($n = \mathrm{h, e, e}$ for n-type trions), $d^{(n)}_{\mathrm{avg,t}} = \sum_l \sigma_{l_n} |w_l|^2 d/2$, and for the free electron, $d_{\mathrm{avg,e}} = \sum_{l_{\mathrm{e}}} \sigma_{l_{\mathrm{e}}} |B_{l_{\mathrm{e}}}|^2 d/2$, where $\sigma_{\mathrm{top}} = +1, \sigma_{\mathrm{bottom}} = -1$, w_l ($B_{l_{\mathrm{e}}}$) is the trion (free electron) layer mixing coefficient, and d the layer separation. When the Coulomb interaction is sufficiently weak (so that it does not induce a significant mixing of the single-particle layer-hybridized bands, as is the case in our work), the average position of the non-recombining electron in the trion ($d^{(\mathrm{e})}_{\mathrm{avg,t}}$) and the free electron ($d_{\mathrm{avg,e}}$) are similar. As a direct consequence, their respective Stark shifts approximately cancel out and the trion resonances shift as $\hbar\omega \to \hbar\omega - e_0 d_{\mathrm{eh}}\varepsilon$, with the dipole moment of the recombining electron-hole pair, $d_{\mathrm{eh}} = d^{(\mathrm{h})}_{\mathrm{avg,t}} - d^{(\mathrm{e})}_{\mathrm{avg,t}}$.

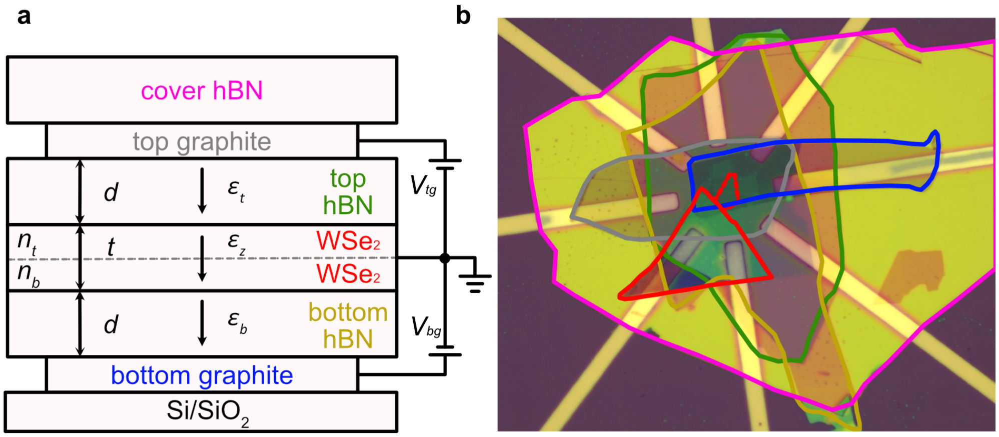

FIG. S1. **Schematics (a) and optical image (b) of the dual-gate bilayer WSe_2 device.** The device consists of six layers on a silicon substrate with a 285 nm thick SiO_2 coating. Two few-layer graphite flakes serve as gate electrodes at voltages V_{tg} and V_{bg}. Two hBN layers are used as dielectrics to separate the WSe_2 bilayer from the gates and a third one is used to cover the device. The contours of the flakes are color-coded into the optical image to indicate their position. The WSe_2 bilayer area is marked with dashed lines. The thickness of the bottom and top hBN was determined by an atomic force microscope ($d = 32\,\mathrm{nm}$). By tuning the voltages V_{tg} and V_{bg}, the out-of-plane electric field ϵ_z in the bilayer and the charge carrier densities n_t and n_b can be controlled. The electric field is defined to point downwards if positive.

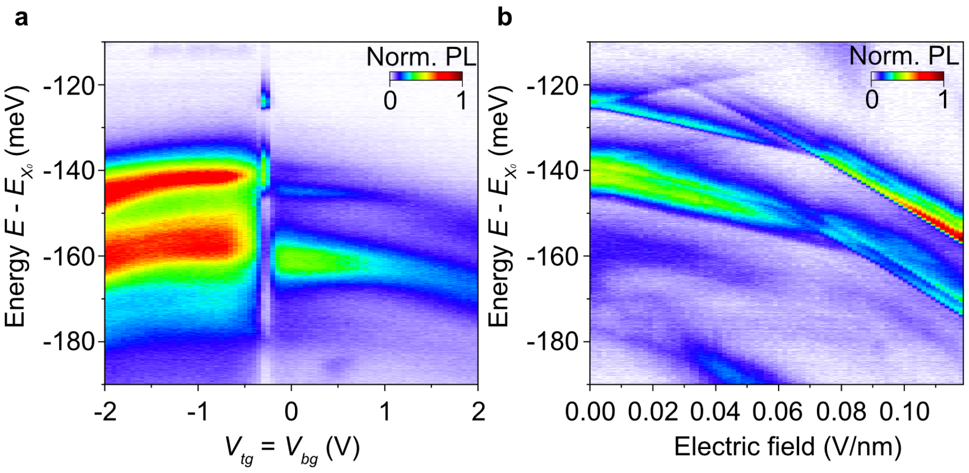

FIG. S2. **Doping dependence of IX PL and electric field dependence of neutral IX PL. a:** Dependence of the interlayer exciton (IX) PL spectrum on the gate voltages V_{tg} and V_{bg}, where V_{tg} is set equal to V_{bg}. Positive (negative) values are associated with an accumulation of electrons (holes) in the sample. **b:** Dependence of the neutral interlayer exciton PL spectrum on an out-of-plane electric field. The electric field is calculated from $\epsilon_z = 3.4(V_{tg} - V_{\mathrm{bg}})/(7.2 \times 2d_{\mathrm{hBN}})$, where 3.4 and 7.2 are the out-of-plane dielectric constants of hBN and WSe_2, respectively.

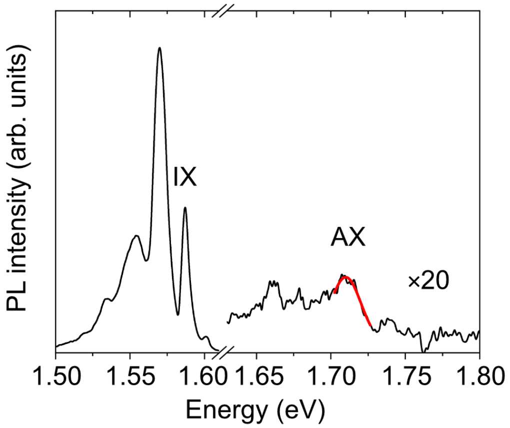

FIG. S3. **PL spectrum of the band-edge excitons.** PL spectrum of natural bilayer WSe_2 when excited by a continuous-wave laser at 488 nm. The AX peak is fitted with a Gaussian function (red line). It is located at 1.71 eV. The interlayer excitons (IX) studied in this work are located more than 100 meV below the AX.

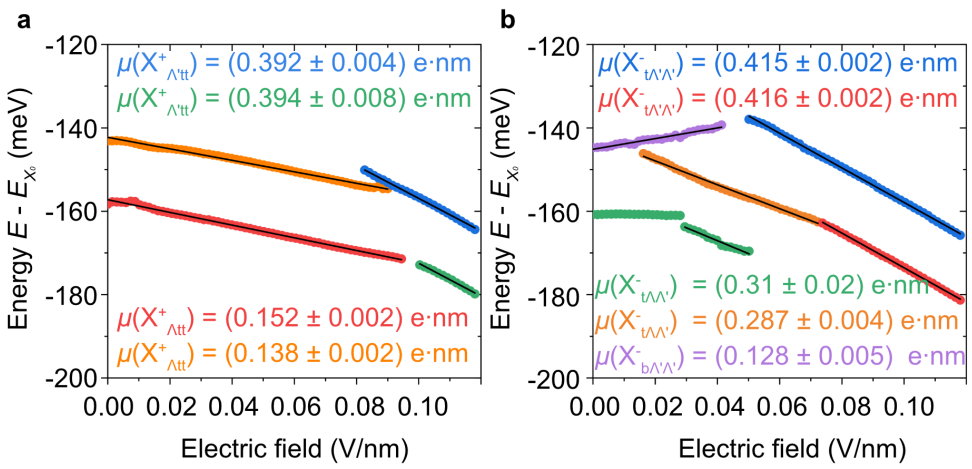

FIG. S4. **Determination of the dipole moments from the experimental data shown in FIG. 4a (a) and FIG. 4b (b) of the main text.** The data points are obtained from Gaussian fits of the peaks in the measured spectra. The dipole moments μ are obtained from this data by a linear fit of the energy shift with respect to the electric field. The relation between the data points and dipole moments is indicated by the color coding. The error values result from the fitting procedure.

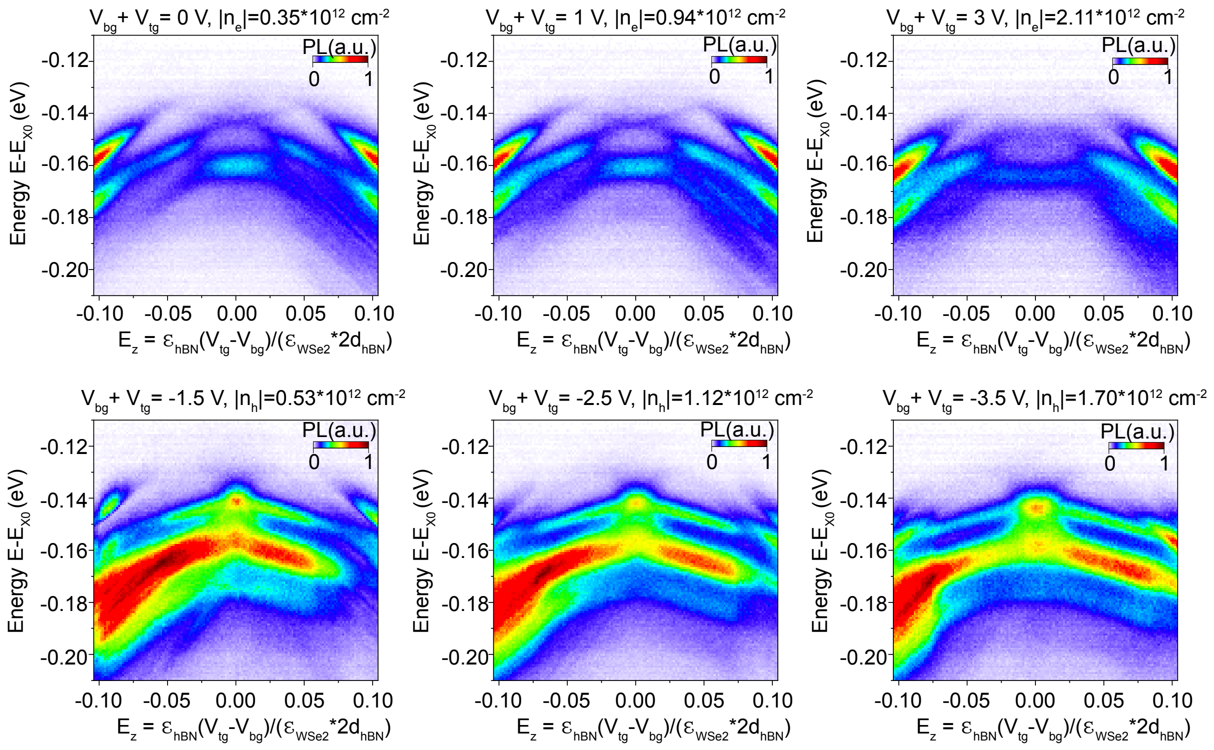

FIG. S5. **Doping dependence of the electric field tunability of trion PL spectra.** Electric-field maps of the trion PL spectra at different n-type (top) and p-type (bottom) doping levels. The doping density is estimated via $n = (c_{\rm tg}V_{\rm tg} + c_{\rm bg}V_{\rm bg})/e_0$, where $c_{\rm bg(tg)} = \epsilon_0\epsilon_{\rm hBN}/d_{\rm hBN}$ is the bottom (top) geometric capacitance, and $d_{\rm hBN} = 32$ nm is the thickness of the bottom (top) hBN sheet. The peak emerging at high energies for low p-type doping at large electric fields (see bottom left panel) is attributed to neutral excitons.

[1] F. Katsch, M. Selig, A. Carmele, and A. Knorr, physica status solidi (b) **255**, 1800185 (2018).

[2] R. Perea-Causin, S. Brem, O. Schmidt, and E. Malic, Physical Review Letters **132**, 036903 (2024).

[3] J. Hagel, S. Brem, C. Linderälv, P. Erhart, and E. Malic, Physical Review Research **3**, 043217 (2021).

[4] A. Laturia, M. L. Van de Put, and W. G. Vandenberghe, npj 2D Materials and Applications **2**, 6 (2018).

[5] N. Rytova, Moscow University Physics Bulletin **3**, 18 (1967).

[6] L. V. Keldysh, Soviet Journal of Experimental and Theoretical Physics Letters **29**, 658 (1979).

[7] D. Erkensten, S. Brem, R. Perea-Causín, and E. Malic, Physical Review Materials **6**, 094006 (2022).

[8] R. Geick, C. Perry, and G. Rupprecht, Physical Review **146**, 543 (1966).

[9] C. Fey, P. Schmelcher, A. Imamoglu, and R. Schmidt, Physical Review B **101**, 195417 (2020).

[10] S. Brem, J. Zipfel, M. Selig, A. Raja, L. Waldecker, J. D. Ziegler, T. Taniguchi, K. Watanabe, A. Chernikov, and E. Malic, Nanoscale **11**, 12381 (2019).

[11] D. Xiao, G.-B. Liu, W. Feng, X. Xu, and W. Yao, Physical Review Letters **108**, 196802 (2012).

[12] J. S. Ross, P. Rivera, J. Schaibley, E. Lee-Wong, H. Yu, T. Taniguchi, K. Watanabe, J. Yan, D. Mandrus, D. Cobden, W. Yao, and X. Xu, Nano letters **17**, 638 (2017).

[13] Z. Wang, Y.-H. Chiu, K. Honz, K. F. Mak, and J. Shan, Nano Letters **18**, 137 (2018).

[14] R. Rosati, K. Wagner, S. Brem, R. Perea-Causin, E. Wietek, J. Zipfel, J. D. Ziegler, M. Selig, T. Taniguchi, K. Watanabe, A. Knorr, A. Chernikov, and E. Malic, ACS Photonics **7**, 2756 (2020).

[15] K.-Q. Lin, C. S. Ong, S. Bange, P. E. Faria Junior, B. Peng, J. D. Ziegler, J. Zipfel, C. Bäuml, N. Paradiso, K. Watanabe, T. Taniguchi, C. Strunk, B. Monserrat, J. Fabian, A. Chernikov, D. Y. Qiu, S. G. Louie, and J. M. Lupton, Nature Communications **12**, 5500 (2021).

[16] Z. Jin, X. Li, J. T. Mullen, and K. W. Kim, Physical Review B **90**, 045422 (2014).

[17] S. Brem, A. Ekman, D. Christiansen, F. Katsch, M. Selig, C. Robert, X. Marie, B. Urbaszek, A. Knorr, and E. Malic, Nano Letters **20**, 2849 (2020).

[18] M. Kira and S. W. Koch, Progress in Quantum Electronics **30**, 155 (2006).

[19] A. Esser, E. Runge, R. Zimmermann, and W. Langbein, Physical Review B **62**, 8232 (2000).

[20] J. Zipfel, K. Wagner, M. A. Semina, J. D. Ziegler, T. Taniguchi, K. Watanabe, M. M. Glazov, and A. Chernikov, Physical Review B **105**, 075311 (2022).

[21] G. Meneghini, M. Reutzel, S. Mathias, S. Brem, and E. Malic, ACS Photonics **10**, 3570 (2023).

[22] S. Brem, K.-Q. Lin, R. Gillen, J. M. Bauer, J. Maultzsch, J. M. Lupton, and E. Malic, Nanoscale **12**, 11088 (2020).

[23] R. Perea-Causin, S. Brem, and E. Malic, Physical Review B **106**, 115407 (2022).

[24] J. Förste, N. V. Tepliakov, S. Y. Kruchinin, J. Lindlau, V. Funk, M. Förg, K. Watanabe,

T. Taniguchi, A. S. Baimuratov, and A. Högele, Nature Communications **11**, 4539 (2020).

[25] J. Zipfel, M. Kulig, R. Perea-Causín, S. Brem, J. D. Ziegler, R. Rosati, T. Taniguchi, K. Watanabe, M. M. Glazov, E. Malic, and A. Chernikov, Physical Review B **101**, 115430 (2020).

[26] M. T. Quick, S. Ayari, N. Owschimikow, S. Jaziri, and A. W. Achtstein, ACS Applied Nano Materials **5**, 8306 (2022).
